# Enhancing steel properties through *in situ* formation of ultrahard ceramic surface

**DOI:** 10.1038/srep38740

**Published:** 2016-12-08

**Authors:** Farshid Pahlevani, Rahul Kumar, Narjes Gorjizadeh, Rumana Hossain, Sagar T Cholake, Karen Privat, Veena Sahajwalla

**Affiliations:** 1 Centre for Sustainable Materials Research and Technology (SMaRT), School of Materials Science and Engineering, UNSW Australia, Sydney, NSW 2052, Australia; 2 Electron Microscopy Unit, Mark Wainwright Analytical Centre, UNSW Australia

## Abstract

Abrasion and corrosion resistant steel has attracted considerable interest for industrial application as a means of minimising the costs associated with product/component failures and/or short replacement cycles. These classes of steels contain alloying elements that increase their resistance to abrasion and corrosion. Their benefits, however, currently come at a potentially prohibitive cost; such high performance steel products are both more technically challenging and more expensive to produce. Although these methods have proven effective in improving the performance of more expensive, high-grade steel components, they are not economically viable for relatively low cost steel products. New options are needed. In this study, a complex industrial waste stream has been transformed *in situ* via precisely controlled high temperature reactions to produce an ultrahard ceramic surface on steel. This innovative ultrahard ceramic surface increases both the hardness and compressive strength of the steel. Furthermore, by modifying the composition of the waste input and the processing parameters, the ceramic surface can be effectively customised to match the intended application of the steel. This economical new approach marries industry demands for more cost-effective, durable steel products with global imperatives to address resource depletion and environmental degradation through the recovery of resources from waste.

Ferrous metal parts used in industry are susceptible to rapid wear and corrosion, which raise significantly the costs of scheduled and unplanned maintenance^1^,^2^,^3^. Increasing the wear and corrosion resistance of ferrous metals extends the life of ferrous metal parts and therefore reduces both replacement and maintenance costs^4^. In recent decades, several methods have been developed to improve the wear and corrosion resistance of ferrous metals. Although these methods have delivered the desired improvements, they are not cost effective, particularly as they require precision equipment and additional processing steps to produce high-grade components. To date, no cost-effective process to produce high wear and corrosion resistant ferrous metals for use in the manufacture of commodity steel products and associated parts has been developed. In recent studies on improving the wear and corrosion resistance of ferrous metals, two main approaches have been employed: 1) enhancing the resistance of ferrous metals in their bulk solid form through microstructure modifications, i.e. heat treatment, the dispersion of the hard phase in a ferrous metal matrix composite and by adding alloying elements; 2) improving wear and corrosion resistance via surface engineering such as the application of coatings, films and surface treatments. Through heat treatment, the wear- and corrosion-resistance properties of ferrous metals can be improved by changing the microstructur^5^,^6^, by low-temperature carburizing^7^, or by introducing a duplex microstructure by controlling the conditions of heat treatment^8^. These processes are effective in improving wear and corrosion resistance, but they have limitations. Consequently, there is significant unmet industry demand for further methods of surface modification to improve the wear and corrosion resistance of steel without affecting its bulk properties. Adding alloying elements can improve the mechanical properties and wear and corrosion resistance of ferrous metals by changing the morphology and the quantities of carbide and graphite in the ferrous metal structure^9^,^10^,^11^,^12^.

In terms of coatings, a low-alloyed ferrous metal substrate and a high-alloyed coating produce the most significant improvement in the wear and corrosion resistance of ferrous metals^13^,^14^. Common fabrication methods for applying such coatings are composite casting^15^, deposit welding^16^ or hot isostatic pressing (HIP) cladding^14^,^17^. Composite casting and deposit welding are quite fast and cheap compared with other coating methods, but suffer from thermodynamic restrictions and an inhomogeneous hard-phase distribution compared with coatings obtained from HIP cladding. Several investigations have shown that HIP cladding can be used to produce high-alloyed coatings^14^,^17^. However, due to the complex encapsulation involved and the need for special HIP furnaces, HIP has proved to be expensive and inflexible as a process technology.

The aim of this research is to devise a new, more cost-effective process to increase the hardness of steel by producing an ultrahard ceramic surface and hard near-surface structure without affecting its bulk properties. It proposes a flexible, new technology that enables an innovative, environmentally sustainable approach to the production of high-tech, cost-effective green materials. The waste integration approach proposed here produces, from waste as raw materials, a ceramic surface chemically bonded *in situ* onto carbon steel to create new steel products with enhanced properties and, consequently, extended useful lives.

The waste input is sourced from end-of-life vehicles. Automotive Shredder Residue (ASR) generally consists of materials such as plastic, rubber, wood, fabric, non-ferrous metals, leather, glass, paper, textiles and dirt^18^,^19^,^20^. Dumping these materials ignores the potential of ASR as a source of important elements, particularly carbon, nitrogen, silicon, aluminum and titanium. While these elements are useful for many applications, the specific focus of this study is their potential for forming a ceramic surface on steel.

This study uses the elements present in ASR to produce an ultrahard ceramic surface, the first such *in situ*, single step process we know of. This highly original process offers significant advantages over conventional approaches. By leverage *in situ* reactions within the existing single heat treatment processes currently used to produce steel products, this approach avoids the need for additional processing steps and, so, avoids additional costs. By utilising waste as an input, costs are, likewise, reduced by avoiding the need for new resources. Beyond these obvious cost and environmental advantages, lie the advantages of flexibility. The composition of the bonded ceramic surface is determined by the nature of the waste stream; as such, the waste input can be modified to suit the intended application of the ceramic surfaced steel and near-surface structure of steel. At the same time by precisely controlling the processing temperatures and reaction duration, the thickness of the ceramic surface can be controlled, as can its properties.

The research study is both economically and environmentally significant. It transforms a problematic global waste stream into resources for production, thereby promoting sustainable manufacturing and reducing the volume of waste going to landfill.

## Method

### Experimental procedure

To investigate the formation of a ceramic surface on steel using ASR we used 0.4% C steel 4mm in diameter and 10mm in length and ASR with an average size of 2mm and a chemical composition as indicated in Table 1. The elemental analyses of ASR and its residue after heat treatment were performed using PerkinElmer quadrupole Nexion Inductively coupled plasma mass spectrometry (ICPMS) integrated with an ESI-New Wave NWR213 Laser Ablation accessory (LA-LCPMS). LECO Tru-Spec analyser was used to determine C, N percentages. The steel samples were put into a zirconia crucible, then filled and covered with raw ASR granules until the crucible was packed tightly. The crucible was then covered with a lid (of the same material as the crucible), to create a closed chamber for the reaction. High purity argon gas was passed through the furnace at a rate of 1 L/min to create the required inert conditions. The crucible was then placed on a graphite rod and fed into the cold zone of the furnace, at a temperature of about 250–300 °C, and kept there for about 10 minutes to prevent thermal shock. It was then pushed into the hot zone which was heated to 1200 °C. Exposure to the high temperature environment in the hot zone was variously 10, 20, 30 and 60 minutes. Once the allotted time had elapsed, samples were pulled back into the cold zone where they were kept for a further 15 minutes to prevent thermal cracking and the oxidation of the steel samples. This temperature has been chosen to be high enough for maximum diffusion rate and at the same time prevent localise melting in steel.

The heat-treated steel samples were then cut using a diamond cutter (Struers Minitom) and prepared for microstructural investigation using a standard sample-preparation technique. The formation of different surface components was investigated with X-ray photoelectron spectroscopy (XPS) using an ESCALAB 250Xi, a high-intensity XPS instrument which uses mono-chromated Al radiation (Ka HV = 1486.7 eV). Orientation microscopy investigation was conducted by electron back-scattered diffraction (EBSD) using an Oxford system attached to a Carl Zeiss AURIGA^®^ field emission gun scanning electron microscope (FEG SEM) workstation to investigate the presence of ceramic surface on heat treated samples. To optimise the EBSD pattern, 2 × 2 binning mode was used with a step size of 0.1 μm/s. The C, N, Ti, Fe, Mn, Al and Si elemental concentration from the ceramic surface into the steel structure was measured by electron probe microanalysis (WDS, JEOL JXA-8500F). EPMA elemental maps were acquired of an area encompassing the coasting and sub-surface at a beam energy of 10 kV, 40 nA current with a dwell time of 30 ms per pixel and a step size of 0.3 μm. A nano-indentation test was conducted using a Hysitron instrument equipped with Tribo Scan analysis software. A maximum load of 5000 μN/sec with a loading and unloading rate of 500 μN/sec and dwell time of 5 secs was used. For the compression test an Instron 5982 equipped with BlueHill 3 analysis software and a 100 kN load cell and a loading rate of 0.5 mm/min was used.

### Theoretical method

Molecular dynamics (MD) simulations using an NVT ensemble with a Nosé-Hoover thermostat were performed within the spin-polarized density functional theory (DFT)^21^ with generalised gradient approximation (GGA)^22^, with projector augmented-wave (PAW) pseudo-potentials, as implemented in the Vienna *Ab-initio* Simulation Package (VASP)^23^. The cut-off energy for the plane-wave expansion was set to be 400 eV. As Fe has a FCC structure at 1200 °C, we considered a FCC structured Fe lattice for MD simulations. The equilibrium lattice constant of the FCC Fe lattice was determined to be 3.45 Å and a thermal expansion of 0.1 Å was considered in the lattice constant at 1200 °C. Therefore, the MD simulation was performed with a lattice constant of 3.55 Å. For the MD simulation, we used a slab of FCC Fe(100) surface with dimensions of 10.65 Å in the *x* and *y* directions and four atomic layers in the *z* direction in a supercell with periodic boundary conditions. One aluminium, one carbon and one oxygen atom were placed on the surface of the slab. We used a vacuum of 10 Å height in the supercell to prevent interactions in adjacent supercells in the *z* direction. The time step for the trajectory was set to 0.5 fs and the simulation was run for 7 ps at 1200 °C. The molecular geometries were recorded every 25 fs, and the results were then used to take the average of the Al-O bond length and distance of C from the Fe surface.

## Results and Discussion

### Formation of ceramic surface

**Figure 1:**
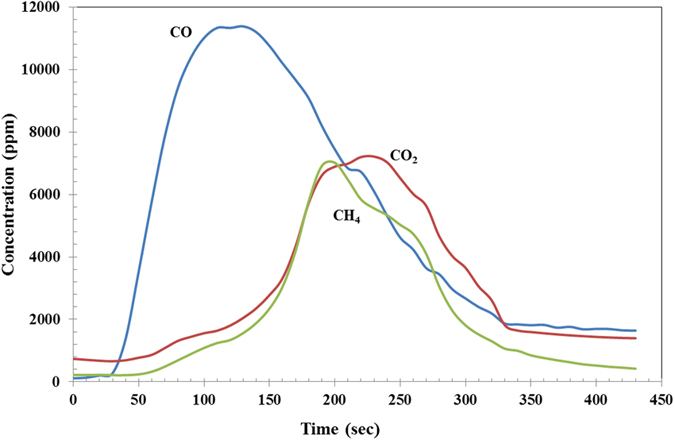
Off gas analysis of heat-treated ASR at 1200 °C.

During the heat treatment of steel with ASR, it was observed that the organic materials in the ASR began to degrade and carbon-saturated gas was produced as indicated in Fig. 1. During this heat treatment, the C-C bond in the organic materials began to break down and the carbon reacted with the oxygen in titanium oxide and silicon oxide to form CO and CO_2_. In general, three main phenomena were occurring on the steel surface; the melting of the existing aluminium and its reaction with oxygen to form aluminium oxide, the conversion of titanium oxide to titanium nitride, the reduction of silicon oxide and the formation of silicon nitride. Steel is a catalyst for all these reactions. As a result, the reduction of titanium oxide and silicon oxide and the formation of titanium and silicon nitride occur at a temperature lower than would be expected for the formation of nitrides. Also, at the same time, carbon from ASR will diffuse into the steel structure and react with the existing Mn in the steel structure and manganese carbide will form.

#### Aluminium oxide

**Figure 2:**
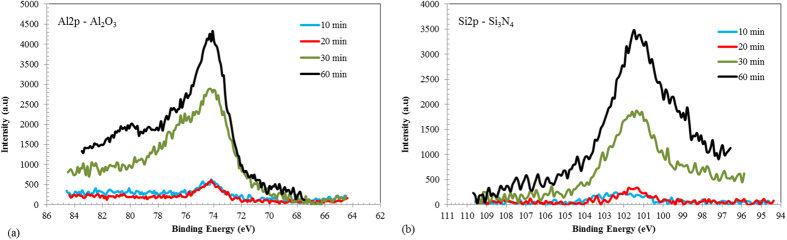
XPS spectra on the surface of steel at different heat treatment times a- Spectra of Al2p which shows the formation of Al_2_O_3_ b- spectra of Si2p which show the formation of Si_3_N_4._

ASR contains small amounts of aluminium which, at 1200 °C, is in a liquid stage. Due to the good chemical bond between the structure of aluminium and iron and the low wettability angle between aluminium and steel^24^, it covers the steel surface. On the other hand, aluminium has a very strong chemical affinity for oxygen and bonds easily with existing oxygen to form aluminium oxide on the steel structure. As this is an exothermic reaction, it will release energy and form local micro-reactors which encourage the formation of aluminium oxide at neighbouring sites. The XPS spectrum of Al2p in Fig. 2a shows the formation of this aluminium oxide surface at different heat treatment times. At longer reaction times the intensity of Al2p increases, which produces an increase in the thickness of the aluminium oxide surface.

**Figure 3:**
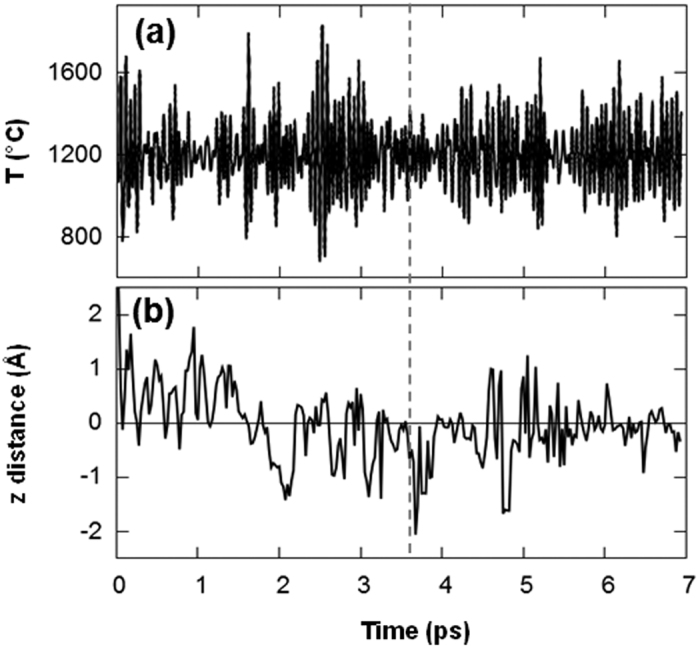
(**a**) Variation of temperature during MD simulation and (**b**) z distance of C from its nearest Fe atom on the surface. The vertical dashed line denotes formation of Al-O at 3.6 ps. A snapshot of the configuration at 3.6 ps is shown in Fig. 4(a,b).

**Figure 4:**
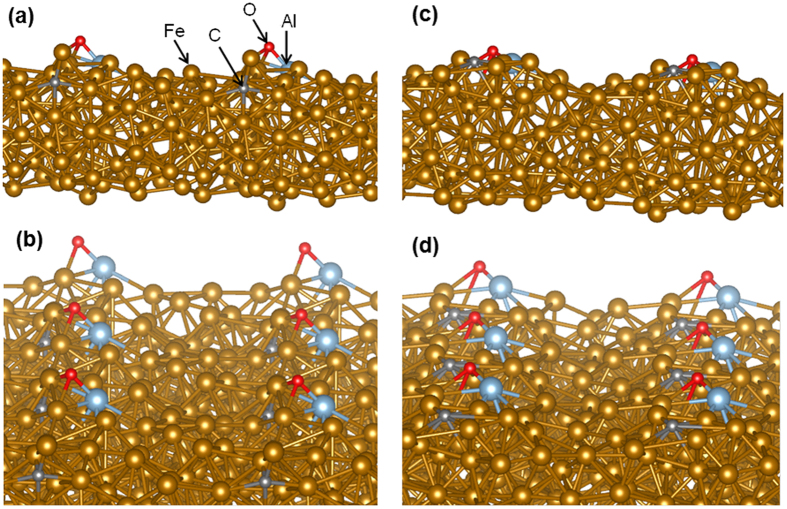
Configuration of 1423 K NVT-MD simulation of FCC Fe(100) with C, Al, and O atoms after (**a,b**) 3.6 ps and (**c,d**) 6.2 ps. (**a**,**c**) show the side view while (**b**) and (**d**) show the top-side view. The configurations depict 6 supercells to show the relative position and distance of C and Al-O from the adjacent atoms. The figure was drawn using Vesta software^26^.

MD simulation confirms that Al-O is formed on the Fe surface at 1200 °C. Figure 3(a) shows the temperature variation during the simulation period for 7 ps. The vertical dashed line at 3.6 ps represents the time that the formation of Al-O starts at the Fe surface. A snapshot of the configuration at 3.6 ps is depicted in Fig. 4(a, b), which confirms that the Al-O bond is formed on the surface while carbon is just below the surface. All the configurations after 3.6 ps up until the end of the MD simulation demonstrate that the Al-O on the surface is stable and forms a strong chemical bond with the Fe surface. The average bond length for Al-O is 1.87 Å, which is in close agreement with experimental results for the bond length of Al-O in the structure of aluminium oxide^25^.

On the other hand, the simulation shows that at this early stage carbon does not diffuse into the Fe and remains just below the surface, close to Al-O on the surface. Figure 3(b) shows the *z* distance of carbon from its nearest Fe atom on the surface. This plot indicates that carbon starts diffusing below the Fe surface before the formation of Al-O occurs on the surface and remains near the surface for the entire simulation time. Figure 4(c,d) shows a snapshot of the configuration at 6.2 ps. As can be noticed from this plot, after the formation of Al-O on the surface, carbon remains close to the Al-O. This implies that there is an attractive interaction between carbon and the Al-O on the surface, which bonds carbon to the surface.

#### Silicon nitride

As well as carbon and nitrogen, ASR contains silicon in the form of SiO_2_, due to the presence of glass in the shredded waste mix. At 1200 °C the reaction between the silicon oxide, reducing gases and carbon residue from the degradation of organic components of ASR will lead to the reduction of SiO_2_. During the process of SiO_2_ reduction, the presence of nitrogen from plastic leads to the formation of silicon nitride as indicated in the equations 1 and 2^26^,^27^. This enables the formation of silicon nitride (Si_3_N_4_) on the surface of the steel. The evidence for this is seen clearly in Fig. 2b, which shows the XPS spectra of the Si2p results for the samples. Generally, the formation of silicon nitride needs a higher temperature and longer exposure time, but in this study iron acts as a catalyst to promote the formation of silicon nitride at a lower temperature and Ar acts as a carrier gas in these reactions^27^. However, compared with aluminium oxide, silicon nitride needs a longer reaction time to form; after 30 minutes the intensity of Si_3_N_4_ in the XPS spectra starts to increase.


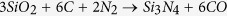



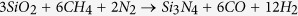


#### Titanium nitride

**Figure 5:**
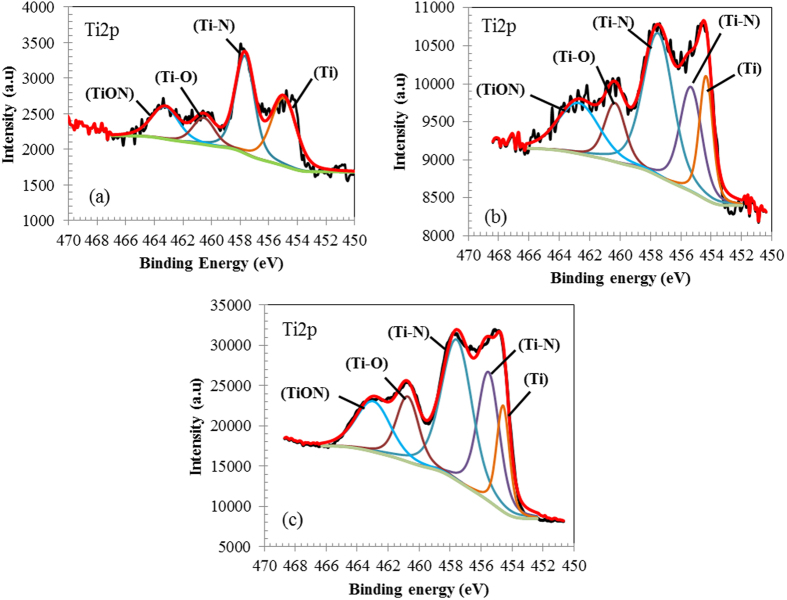
XPS spectra of steel surface heat treated at different times: a-20 min, b-30 min and c-60 min.

Another important component in ASR is titanium oxide which is derived from titanium oxide pigment in the colours as well as the UV stabiliser in the plastics. Recent studies have shown that the reduction of titanium oxide in ASR by carbon from degraded organic components has been followed by the nitridation of Ti to form TiN^28^,^29^. This transformation of titanium oxide to titanium nitride will take place during the nitridation process as indicated in equations 3 and 4. The XPS spectra of Ti2p on the steel surface at different heat treatment times (Fig. 5) show the formation of the Ti-N bond and transference on the Ti-O bond to Ti and then to a Ti-N bond.


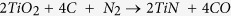



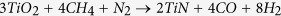


Table 2 summarises the formation of the chemically-bonded ceramic surface on steel at different heat treatment times. As the table shows, the first ceramic surface which forms on the steel surface from 10 minutes is aluminium oxide because aluminium is in a liquid stage at 1200 °C and the reaction kinetic is fast. After 20 minutes a titanium nitride surface starts to form and after 30 minutes a silicon nitride surface appears. A recent study has shown that hydrogen will accelerate the reduction of silicon oxide and titanium oxide and iron will work as a catalyst in the formation of different ceramic components. Given the small diameter of hydrogen atoms and their highly reactive nature, in particular with oxygen, the presence of hydrogen in the system increases the reduction speed of oxides^27^. In the present study, hydrogen from the degradation of organic components helps in reducing the oxide phases and, because of this reaction, there is no free hydrogen to diffuse into steel and cause a hydrogen embrittlement effect. All these reactions which form ceramic layers occur on the steel surface, which increases the yield of ceramic surface formation by enhancing the rate of reduction and nitridation^30^.

**Figure 6:**
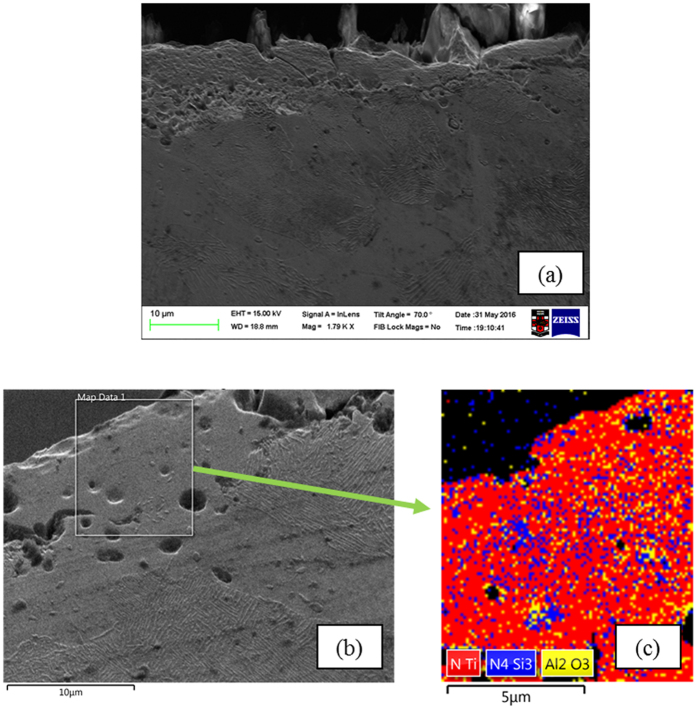
SEM and EBSD Phase maps of 30 min treated sample. (**a**) SEM image of chemically bonded ceramic surface on steel. (**b**) Selected area for EBSD phase map analyse. (**c**) Combined EBSD phase map of all phases, different colours characterise different elements, TiN is red, Al_2_O_3_ is yellow, Si_3_N_4_ is blue.

The cross-section of a sample heat treated at 1200 °C for 60 minutes was investigated using the SEM and EBSD micrograph to identify the morphology of different ceramic phases on the sample’s surface. As shown in Fig. 6a, an ultrahard ceramic layer has formed on the steel surface and, according to the EBSD analyses, which identify the crystallographic information and orientation of the grains and has been shown in Fig. 6b and 6c, this ultrahard ceramic surface is the combination of TiN, Al_2_O_3_ and Si_3_N_4_ phases, as these ceramic phases form simultaneously. These ceramic phases formed on the steel surface increase its hardness and, as they are chemically-bonded to the steel surface, they will resist applied force better than physically bonded ceramic surfaces.

**Figure 7:**
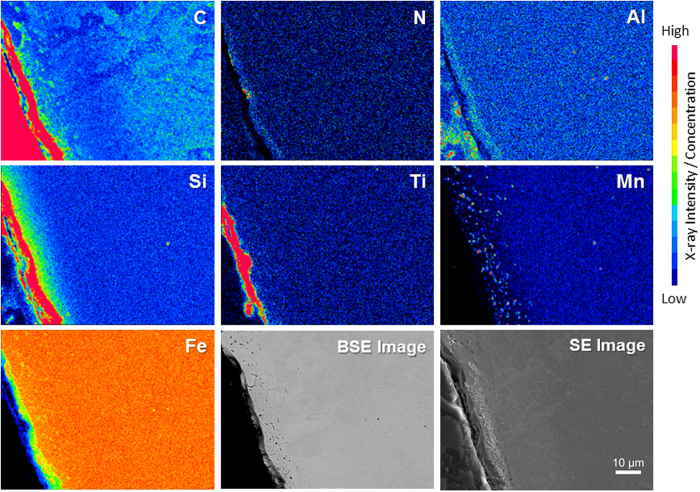
EPMA X-ray intensity map for C, N, Ti, Fe, Mn, Al and Si Kα at the steel surface and near-surface region showing the relative concentration of these elements. Blue is lower concentration and Red higher concentration. Scale bar = 10 μm.

Figure 7 shows the EPMA results for the distribution of C, N, Ti, Fe, Mn, Al and Si from the ceramic surface to the bulk steel structure. SEM and EPMA results reveal the structural continuity of the ceramic surface and steel substrate, indicating that the ceramic surface has been grown from ASR and chemically bonded to the steel surface. Due to the larger amount of Si in the ASR the silicon nitride, which is in combination with silicon carbide layer, is thicker than the titanium nitride layer. There is a diffusion of these elements into the steel’s structure as it can be seen from the gradient of the elements’ concentrations in Fig. 7 and all the reactions have occurred on the steel surface. Carbon and manganese maps show that by increasing the heat treatment time, carbon starts to diffuse into the steel and react with Mn in the steel structure, forming manganese carbide. These results show that at the early stage of heat treatment carbon atoms are bonded to the surface by the formation of an Al-O bond but as heat treatment time increases carbon starts to diffuse into the steel and carbide phases will be formed. EPMA mapping clearly indicated that a chemical-bonded ceramic surface is formed on the steel surface and, by diffusion of carbon, sub-micron carbide phases will form near the surface region, increasing the hardness of the surface.

### Mechanical properties

**Figure 8:**
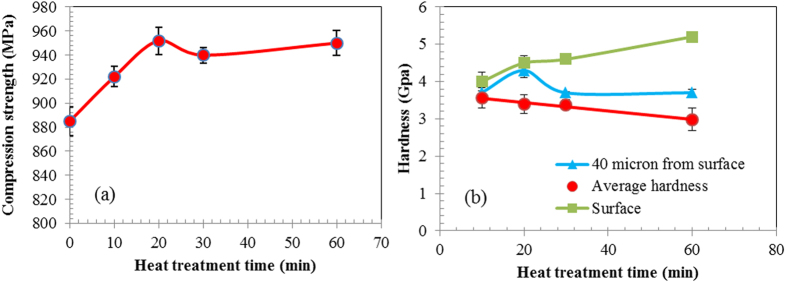
(**a**) Compression strength and (**b**) hardness of steel after formation of ceramic surface.

After heat treatments of different durations, the samples were subjected to compression tests to identify the effect of both the resulting ceramic surface and heat treatment on the compressive strength of the steel. Fig. 8a and Table 3 shows the variations in the compressive strength of the steel samples after heat treatments ranging from 0 to 60 minutes. The compressive strength of the steel is believed to be a comprehensive outcome resulting from the combined effects of the formation of the hard surface and grain size. After heat treatment, the harder surface – the ceramic phase – was observed to increase compressive strength, but the increase in the grain size due to longer heat treatment was observed to reduce compressive strength. In Fig. 8a, the initial portion of the graph (from 0 to 20 min) shows a gradual increase in compressive strength that indicates the effect of the hard surface outweighs, or dominates, the effect of an increased grain size. At 30 minutes of heat treatment, however, grain size growth emerges and dominant and the net result is that no further increase in strength is observed from 20 to 30 minutes heat treatment time. By increasing the heat treatment time to 60 minutes, thereby increasing carbon diffusion into the steel, the formation of carbide phases near the surface is increased, resulting in a thicker ceramic surface and an increase in strength.

To analyse the effect of the ceramic surface as well as the increase in grain size due to the increase in heat treatment time, the hardness of the samples was measured at different positions using nano-indentation. Fig. 8b shows the surface hardness of the samples, the hardness at 40 microns from the surface, as well as the average hardness of the samples at the centre. Fig. 8b also shows that increased grain size cause small reduction in the average hardness of the steel at its centre. However, increasing heat time increases the thickness of the ceramic surface as well as diffusion of carbon into the steel and the formation of the sub-micron manganese carbide phase, and therefore an increase in the steel’s surface hardness, as shown in Fig. 8b. This indicates the need to optimise the product’s strength by balancing gains in surface hardness due to longer heat times against potential losses in compression strength due to grain size increases, or by pinning the grains using a secondary phase to avoid grain growth due to heat treatment.

By increasing the heat treatment time, the thickness of the ceramic surface increases and both the diffusion of carbon into the steel structure and the formation of the manganese carbide phase are initiated; increasing the hardness of steel surface as indicated in Fig. 8b. By increasing the heat treatment time, the concentration of diffused carbon and its diffusion depth will change and, at the same time, manganese carbides’ size increase and their population start to decrease. This results in decreasing the hardness at 40 micron from surface after between 20 minute and 30 minute heat treatment. But, by increasing the heat treatment time from 30 minute to 60 minutes there is small increase in hardness at 40 micron from surface, due to the increase in diffused carbon. These results indicate that by controlling the heat treatment to control the grain size, carbon diffusion as well as thickness of the ceramic surface we can achieve greater gains in hardness and we can tailor the desire mechanical property on the surface, near the surface and at the centre of the steel.

## Conclusions

In this research, the formation of a ceramic surface *in situ* on normal carbon steel by heat treating steel with automotive shredder residue has been investigated. XPS results indicate that after 10 minutes of heat treatment, aluminium oxide is formed on the steel’s surface. After 20 minutes titanium nitride is formed and after 30 minutes silicon nitride is formed on the steel’s surface. From nano-indentation and compression-strength measurements, it is clear that the formation of these *in situ* ceramic surfaces increases the hardness and mechanical strength of normal carbon steel.

These results show the compressive strength of the ceramic-coated steel samples peaked at 20 minutes exposure at 1200 C, before the formation of silicon nitride at 30 minutes exposure. Consequently, the benefits in terms of surface durability of the additional layer of chemically-bonded silicon nitride need to be balanced against a minor reduction in compressive strength, when compared to the additional strength achieved at 20 minutes and, later, at 60 minutes exposure. However, the improved surface hardness resulting from the additional ceramic formation phase at 30 minutes, and a consequently thicker ceramic surface, needs to be taken into account. In addition, by modifying heat treatment conditions, especially by quenching the sample after heat treatment or by extending the heat treatment time to increasing the diffusion of carbon and to promote carbide phase formation near the surface, we can compensate for the reduction in compressive strength and still take advantage of the full benefits of the chemically-bonded ultrahard ceramic surface. It is also important to note that at every exposure time tested between 10 and 60 minutes, the compression strength of the heat treated steel and ASR product was superior to a raw steel sample.

These results highlight the potential use of ASR waste as a valuable resource for production. As such, the use of ASR waste materials to form a ceramic surface on steel is an innovative and effective way to produce steel components with enhanced properties at low cost, while simultaneously reducing demands for raw materials for coatings or alloying, and alleviating the environmental impact of ASR dumped in landfills.

## Additional Information

**How to cite this article**: Pahlevani, F. *et al*. Enhancing steel properties through *in situ* formation of ultrahard ceramic surface. *Sci. Rep.* **6**, 38740; doi: 10.1038/srep38740 (2016).

**Publisher's note:** Springer Nature remains neutral with regard to jurisdictional claims in published maps and institutional affiliations.

## Figures and Tables

**Table 1:** Chemical composition of ASR samples.

Elements	C (wt%)	N (wt%)	Ti (wt%)	Si (wt%)	Al (wt%)
Raw ASR	19.43	0.72	2.68	0.49	0.1
ASR at 1200 °C	61.45	1.4	12.55	5.45	0.45

**Table 2:** Chemical-bonded ceramic on steel surface.

Sample	Ceramic surface		
	Al_2_O_3_	Si_3_N_4_	TiN
1200–10 min	√		
1200–20 min	√		√
1200–30 min	√	√	√
1200–60 min	√	√	√

**Table 3:** Compression strength after formation of ceramic surface.

Sample	Compression strength (MPa)
Raw sample	885
1200–10 min	922
1200–20 min	952
1200–30 min	940
1200–60 min	950
